# Involvement of a Response Regulator VdSsk1 in Stress Response, Melanin Biosynthesis and Full Virulence in *Verticillium dahliae*

**DOI:** 10.3389/fmicb.2019.00606

**Published:** 2019-03-22

**Authors:** Jiayue Zheng, Chen Tang, Chenglin Deng, Yonglin Wang

**Affiliations:** Beijing Key Laboratory for Forest Pest Control, College of Forestry, Beijing Forestry University, Beijing, China

**Keywords:** *Verticillium dahliae*, the two-component system, melanized microsclerotia, virulence, stress response

## Abstract

*Verticillium dahliae* causes vascular wilt disease on over 200 plant species worldwide. This fungus forms melanized microsclerotia which help it to survive under adverse conditions and these structures are vital to the disease spread. Here, we identified and characterized a *V. dahliae* homolog to of the *Saccharomyces cerevisiae* Ssk1, a response regulator of the two-component system. Herein, we demonstrated that the *VdSsk1* deletion strains were more sensitive to various stresses, including oxidative stress conferred by H_2_O_2_ and sodium nitroprusside dihydrate, while the mutants confered higher resistance to fungicides such as fludioxonil and iprodione. Furthermore, disruption of *VdSsk1* resulted in significant downregulation of melanin biosynthesis-related genes but did not affect microsclerotial development. Phosphorylation of VdHog1 was not detected in the *VdSsk1* deletion strains under the treatment of sorbitol, indicating that phosphorylation of VdHog1 is dependent on VdSsk1. Finally, we demonstrated that VdSsk1 is required for full virulence. Taken together, this study suggests that VdSsk1 modulates stress response, melanin biosynthesis and virulence of *V. dahliae*.

## Introduction

The two-component system (TCS) pathway is a primary means of responding to external stimuli, and is widely conserved in bacteria, fungi, and plants (Bourret et al., 1991; Alex et al., 1996). Generally, most eukaryotic TCSs always comprise a membrane-bound sensor histidine kinase (HK), a response regulator (RR) and a His-containing phosphotransfer protein (HPt) (Motoyama et al., 2008). For instance, in the budding yeast *Saccharomyces cerevisiae*, the TCS pathway contains one HK (Sln1), one Hpt protein (Ypd1) and two RRs (Ssk1 and Skn7) (Schaller et al., 2011). The histidine kinase functions as the sensor reacting to external stress signals and activates RRs, which trigger downstream responses including stress signaling pathways and expression of stress response genes. Under conditions of high osmolarity, the RR Ssk1 is dephosphorylated and activates the mitogen-activated protein kinase (MAPK) kinase that initiates the high-osmolarity glycerol (HOG) MAPK pathway for control of the osmotic stress response (Sato et al., 2003). Moreover, RR Skn7 is a transcription factor containing an N-terminal HSF (heat shock factor)-type DNA binding domain, and is phosphorylated by Sln1 to activate hypo-osmotic response genes (Li et al., 2002). Therefore, Ssk1 acts through the HOG MAPK cascade as an upstream regulator in *S. cerevisiae*, while Skn7 functions as a stress response transcription factor to adapt to conditions of high osmolarity.

Although orthologs of Ssk1 are highly conserved among yeasts and filamentous fungi, contradictory roles in the adaption of various stresses are found in several fungi. For example, deletion of *Ssk1* in *Kluyveromyces lactis* does not affect sensitivity to hyperosmotic stress (Rodriguez-Gonzalez et al., 2017). However, disruption of *Ssk1* from *Candida albicans* causes increased sensitivity in the high salinity conditions (Chauhan et al., 2003). Furthermore, in pathogenic fungi, Ssk1 plays roles in fungal virulence (Jiang et al., 2011; Yan et al., 2011; Wang et al., 2014; Yu et al., 2016). For instance, in *Beauveria bassiana*, the virulence and tolerances to osmotic stress are both significantly reduced by the deletion of *Ssk1* (Wang et al., 2014). In vascular wilt fungi, changes in the plant xylem fluids may contribute to decreases in virulence if the pathogen cannot respond adequately to osmotic stress inevitably found in this niche, as previously suggested (Klosterman et al., 2011). Yet the function of Ssk1 in vascular wilt fungi, during colonization, and in response to osmotic stress, has not been investigated.

*Verticillium dahliae* is a notorious vascular pathogen, infecting over 200 plant species, including important cash crops and ornamental plants, i.e., smoke trees (*Cotinus coggygria*), a major component of the scenery of the Fragrance Hills Park in Beijing, China (Wang et al., 2013). There are no effective curative fungicides available to control *V. dahliae* once the plants are infected (Klosterman et al., 2009). Importantly, the fungus also produces melanized dormant resting structures, known as microsclerotia. Microsclerotia can survive in soils without a plant host for more than 10 years and are the primary infectious propagules of the wilt disease (Wilhelm, 1955). *V. dahliae* penetrates its host root as the hyphae which germinate from microsclerotia and colonize the plant xylem vessels. Therefore, microsclerotia play crucial roles in the disease cycle and are considered key targets for disease control (Klosterman et al., 2009; Klimes et al., 2015).

The availability of *V. dahliae* genome sequences has greatly facilitated our capacity to identify and characterize genes involved in microsclerotia formation and stress response. In recent years, dozens of genes involved in signaling transduction have been characterized, including those of MAPK pathways (VMK1, VdMsb, VdSho1, VdPbs2, VdHog1, and VdMsn2) (Rauyaree et al., 2005; Qi et al., 2016; Tian et al., 2016, 2017; Wang et al., 2016) and cAMP-PKA- (VdPKAC1) (Tzima et al., 2010) and G protein (VGB)-mediated signaling (Tzima et al., 2012). Recently, we systematically explored aspects of the HOG-MAPK pathway in *V. dahliae*. Deletion of *VdHog1* and *VdPbs2* resulted in delayed microsclerotia formation, attenuated virulence and enhanced sensitivity to hyperosmotic stress (Tian et al., 2016; Wang et al., 2016). In addition, we found that VdSsk22 acts upstream of the VdPbs2-VdHog1 module and regulates microsclerotial development and stress responses (unpublished data). We have further showed that the two-component response regulator VdSkn7 plays key roles in stress resistance, microsclerotial development, and virulence (Tang et al., 2017). However, it remains to be determined whether the second RR gene, VdSsk1, is involved in the regulation of stress responses and microsclerotia formation in *V. dahliae*.

In this study, we demonstrated that VdSsk1 acts as a major regulator of stress responses and melanin biosynthesis during microsclerotial development in *V. dahliae*. We also revealed that VdSsk1 acts as an upstream of the HOG-MAPK cascade through phosphorylation analyses and plays a crucial role in virulence. These findings indicate that VdSsk1 governs the signaling required for appropriate stress responses, melanin biosynthesis, and for full virulence in *V. dahliae*.

## Materials and Methods

### Fungal Strains and Growth Conditions

The wild type *V. dahliae* strain XS11 (Wang et al., 2013) was isolated from a smoke tree (*Cotinus coggygria*) in the Fragrant Hill Park, Beijing and used for transformation. Both the wild type and transformants were stored at -80°C as conidial suspensions in 30% glycerol. The single-spore isolates were cultured on potato dextrose agar (PDA) medium (200 g potato, 20 g glucose, 20 g agar per liter) at 25^o^C for 7 days. After this period, culture plugs made by 5 mm punches were transferred to liquid complete medium (CM, 50 ml 20 × nitrate salts, 1 ml 1000 × Trace, 10 g glucose, 2 g peptone, 1 g yeast extract, 1 g casamino acids, and 1 ml vitamin solution in one liter) with shaking for 5 days. Vegetative hyphae were collected for genomic DNA extraction. Hygromycin- or geneticin-resistant strains were cultured on PDA amended with 25 μg/ml hygromycin or 50 μg/ml geneticin, respectively.

To test their sensitivity to the cell wall stress, all strains were cultured in solid CM containing 50 μg/ml Congo red (CR) or 10 μg/ml calcofluor white (CFW) for 12 days. For the osmotic stress assays, 0.4 M NaCl, 0.4 M KCl was added in the CM and cultures incubated for 12 days. To test the sensitivity to fungicides, four kinds of fungicides were used: 5 μg/ml difenoconazole, 2 μg/ml chlorothalonil, 10 μg/ml fludioxonil, and 5 μg/ml iprodione. To test sensitivity to oxidative stress, 100 μL 1 × 10^7^ conidial suspensions of each strain were mixed with PDA before pouring into plates with filter paper disks containing a drop of 5 μl 15 or 30% hydrogen peroxide (H_2_O_2_) in the center. As for nitrooxidative stress assay, 10 and 30 mM sodium nitroprusside dihydrate (NPS, Merck) were added to CM.

To discover the differences in microsclerotial formation of all strains, spore suspensions in CM cultured for 5 days, were diluted to 1 × 10^5^. Then conidia were dripped onto the cellulose membrane (Ø = 80 mm; pore size = 0.22 μm) on solid basal medium (BM) containing 10 g glucose, 0.2 g sodium nitrate, 0.52 g KCl, 0.52 g MgSO_4_.7H_2_O, 1.52 g KH_2_PO_4_, 3 μM thiamine HCl, 0.1 μM biotin, and 15 g agar per liter. Microsclerotia formation was examined and imaged under light microscope (Leica DM 2500).

### Bioinformatics Analysis

The protein sequence of VdSsk1 was acquired from Broad Institute *V. dahliae* sequence (Klosterman et al., 2011) maintained by JGI^1^ using Blastp. Multiple sequence alignments were conducted using ClustalX 2.0 and the phylogenetic tree was constructed by MEGA 6.0 with the neighbor-joining algorithm under default settings and 1000 bootstrap replications (Tamura et al., 2013).

### Target Gene Knockout and Complementation

The Δ*VdSsk1* deletion strains were generated using the split-marker method (Goswami, 2012). The primer set Ssk1-5Ffor/Ssk15Frev and Ssk1-3Ffor/Ssk1-3Frev were used to amplify the upstream and downstream flanking sequence, respectively (Supplementary Table 1). Then, the amplified fragments were fused with a geneticin-resistant cassette with primers Ssk1-3Frev/Ge-R and Ssk1-5Ffor/Ge-F. All of fragments mentioned above were confirmed by sequencing. Protoplasts of *V. dahliae* XS11 were transformed with these DNA fragments. The *VdSsk1* deletion strain was selected and examined using PCR with primes Ssk1-Infor/Ssk1-Inrew, Ssk1-Exfor/Ssk1-Exrew, respectively. Finally, the deletion strain was confirmed by Southern blot. For gene complementation, the fragment consisting of the native promoter and the entire open reading frame was amplified from genomic DNA using primes Ssk1-comfor/Ssk1-comprev, then was co-transformed into protoplasts of the Δ*VdSsk1* mutant with a hygromycin resistance cassette for future selection. All the complementation strains were confirmed by PCR.

### RNA Extraction and Reverse Transcription Quantitative PCR

All of the strains including XS11 and Δ*VdSsk1* strains were cultured in CM at 25 C for 5 days, and the mycelia were collected after filtering through a single-layer of miracloth. Total RNA was extracted with Trizol Reagent (Invitrogen, United States) and then further purified with PureLink DNase Kits (Invitrogen, United States). Reverse-transcription PCR was performed using QuantScript RT Kit (TIANGEN, China), then reverse transcription quantitative (qRT-PCR) was performed with SuperReal Premix Plus (SYBR Green) (TIANGEN, China) and ran on an ABI 7500 real-time PCR system (Applied Biosystems, United States). The β-tubulin gene of *V. dahliae* was used as an internal reference, while the relative expression levels of *VdSsk1* were calculated using the ΔΔCT method. All primers used in this study were listed in Supplementary Table 1. These experiments were repeated three times.

### Pathogenicity Assays and Infection Process

Conidial suspensions of all strains were collected from liquid CM medium where the fungus had been cultured for 5 days at 25^o^C, and diluted to 1 × 10^6^ for further use. Forty tobacco plants under the same growth condition were inoculated by soaking the roots in the conidia suspensions for 20 min then planted into the sterilized soil in a greenhouse. After 25 days, all tobacco plants were examined for symptoms and death, including leaves and roots.

To investigate the infection process, onion epidermal strips (kept in 75% ethanol) were placed on water agar (8 g/L agar) plates, with the hydrophobic surface upward. Conidial suspensions were diluted to 1 × 10^5^, and drops of 1 μl were loaded on the surface, and the plates were incubated at 25^o^C for 24 h before examination under light microscope (DM2500, Leica).

Cellophane membranes were placed on the MM (6 g NaNO_3_, 0.52 g KCl, 1.52 g KH_2_PO_4_, 10 g glucose, 0.52 g MgSO_4_⋅7H_2_O, 2.94 g L-glutamic acid, 15 g agar per liter) plates with all strains inoculated on them. After 3 or 5 days, the cellophane membranes were removed and inoculated for another 24 h, then checked whether or not the colonies have developed. The number of hyphopodia and penetration peg were counted under light microscopy. The experiments were repeated three times.

### Immunoblot Analyses

Total proteins were isolated from 48 h liquid CM cultures that consisted of fresh mycelia. The mycelia were transferred to a 1.2 M sorbitol solution for 2 h, then separated by SDS-PAGE using 12.5% gel. Separated total proteins were transferred to a nitrocellulose membrane and the anti-phospho-p38 monoclonal antibody (P-Hog1, Cell Signaling) was used to detect the phosphorylated form of the VdHog1p. Sampling staining was set as the internal control and chemiluminescent detection was performed.

### Statistical Analysis of Melanized Microsclerotia

Melanized area was calculated using ImageJ by adjusting the brightness of the 8-bit format image and measuring the dark area. Data were expressed as the means ± standard errors. Statistical analyses were performed by using the independent-samples *t*-test in SPSS Base 11.0; ^∗^*P* < 0.05 and ^∗∗^*P* < 0.01.

## Results

### Loss of *VdSsk1* Causes Hypersensitivity to Osmotic Stress and Increased Tolerance to Fludioxonil and Iprodione

Previously, we have discovered the main components of TCS encoded in the *V. dahliae* genome (Tang et al., 2017). With the exception of VdSkn7 (*VDAG_04946*), none of the TCS genes were further characterized. Here we focused on VdSsk1 (*VDAG_06915*, Accession No. XP_009652845), a gene sharing homology to the yeast Ssk1. VdSsk1 encodes a protein of 818 amino acids with a REC domain. Phylogenetic analysis demonstrated that VdSsk1 orthologs are highly conserved among fungi (Supplementary Figure S1).

**Figure 1:**
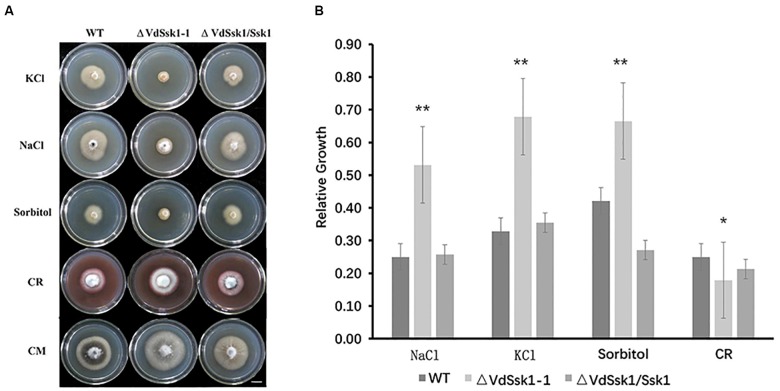
VdSsk1 plays an essential role in osmotic stress resistance in *V. dahliae.* **(A)** Images of the colony morphology of the wild type, Δ*VdSsk1*, and Δ*VdSsk1/Ssk1* strains grown on complete medium (CM) or CM supplemented with 0.4 M NaCl, 0.8 M KCl, 1.2 M sorbitol, and 50 mg/ml Congo red (CR) for 12 days. Scale bar = 1 cm. **(B)** Bar charts show mycelial growth of strains in **(A)** by measuring the diameter of each of the colonies grown on CM with or without treatment. Error bars represent the standard deviation of three replicates and asterisks represent statistical differences (^∗^*P* < 0.05 and ^∗∗^*P* < 0.01) determined by a Student’s *t* test in comparison with the wild type strain.

**Figure 2:**
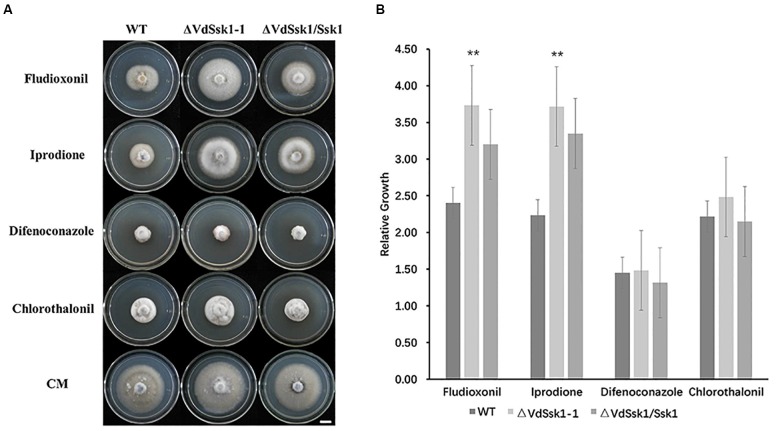
The *VdSsk1* mutants confer resistance to fungicides. **(A)** The growth of the wild type, Δ*VdSsk1*, and Δ*VdSsk1/Ssk1* strains on complete media (CM) amended with 10 μg/ml fludioxonil, 5 μg/ml iprodione, 5 μg/ml difenoconazole, and 2 μg/ml chlorothalonil for 12 days. Scale bar = 1 cm. **(B)** The chart illustrates mycelial growth of strains in **(A)** by measuring the diameter of colonies grown on CM with or without fungicides. Error bars represent the standard deviation of three replicates and two asterisks represent significant differences at *P* < 0.01 revealed by Student’s *t*-test.

To examine the function of VdSsk1, *VdSsk1* deletion mutants were generated by targeted replacement of the wild-type *VdSsk1* with a geneticin resistance cassette. The potential *VdSsk1* deletion strains were screened by genomic PCR and the deletion mutants were confirmed by southern blot (Supplementary Figure S2). In addition, the wild-type *VdSsk1* copy was ectopically reintroduced into the Δ*VdSsk1* deletion strain, yielding the Δ*VdSsk1*/*VdSsk1* complemented strain (Supplementary Figure S2). The Δ*VdSsk1* deletion strain exhibited no obvious growth defect on PDA and CM, but the mycelial growth was obviously reduced in the presence of KCl, NaCl, and sorbitol. Compared with the growth of wild type strain XS11, the Δ*VdSsk1* strain was significantly decreased in radial growth to about 50% of wild type strain XS11 (Figure 1). However, deletion of *VdSsk1* resulted in significantly enhanced resistance to CR (Figure 1). Furthermore, homologs of Ssk1 in other fungi are required for resistance to fludioxonil (Banno et al., 2007; Jones et al., 2007). Expectedly, compared to XS11 and the complemented strain, the Δ*VdSsk1* strain showed significant resistance to fludioxonil and iprodione (Figure 2). However, the Δ*VdSsk1* strain did not exhibit differential resistance or sensitivity to difenoconazole and chlorothalonil in comparison with XS11 and complemented strain (Figure 2). These data indicated that VdSsk1 is involved in the response to various stresses, including high osmolarity stress.

### VdSsk1 Inactivation Increases Sensitivity to Oxidative and Nitrooxidative Stress

**Figure 3:**
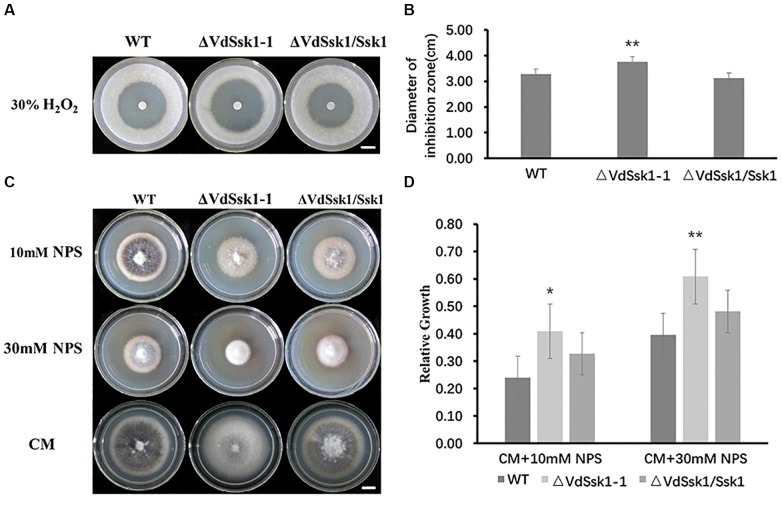
VdSsk1 is required for the tolerance to oxidative and nitrooxidative stress. **(A)** Images of inhibition zones. Conidia at a concentration of 1 × 10^7^ conidia/ml of the wild type, Δ*VdSsk1*, and Δ*VdSsk1/Ssk1* strains were added to potato dextrose agar (PDA) plates in which circular filter paper containing 30% H_2_O_2_ was placed. The plates were incubated at 25°C for 4 days. Scale bar = 1 cm. **(B)** The chart represents the diameter of the suppression zone for the plates above. Error bars represent the standard deviation of three replicates and two asterisks represent significant differences (*P* < 0.01) determined by Student’s *t* test in comparison with the wild type strain. **(C)** The wild type, Δ*VdSsk1*, and Δ*VdSsk1/Ssk1* strains were cultured on CM with 10 or 30 mM sodium nitroprusside dihydrate (NPS) for 12 days. Scale bar = 1 cm. **(D)** The chart shows the relative growth of each strain in **(C)** by measurements of diameters of mycelial colonies grown on CM with or without NPS. Error bars represent the standard deviation of three replicates. Asterisks indicate statistical significance (^∗∗^*P* < 0.01 and ^∗^*P* < 0.05) determined by Student’s *t* test.

We investigated the role of VdSsk1 in conferring the sensitivity to H_2_O_2_. The sensitivity on CM with the addition of H_2_O_2_ revealed that the Δ*VdSsk1* deletion strain was more sensitive to H_2_O_2_. The suppression zone observed for the Δ*VdSsk1* strain was significantly larger than that observed for XS11 and the complemented strain (Figure 3A,B). Because nitric oxide (NO) reacts with reactive oxygen species (ROS) such as the superoxide anion to generate reactive nitrogen species (RNS), we sought to determine whether *VdSsk1* is involved in the nitrooxidative stress as well. The mycelial growth of the Δ*VdSsk1* strain was obviously inhibited by the addition of 10 and 30% sodium nitroprusside dihydrate (NPS), a donor of NO (Figure 3C,D). We further investigated the growth of the Δ*VdSsk1* strain on NO_3_^-^ and NO_2_^-^ media, in which they also generate RNS. Unexpectedly, Supplementary Figure S3 shows that fungal growth of the Δ*VdSsk1* strain was comparable to XS11 and complemented strain, indicating that *VdSsk1* is not required for resistance to RNS produced by the metabolism of NO_3_^-^ and NO_2_^-^. Taken together, these results indicated that VdSsk1 plays an important role in resistance to oxidative and nitrooxidative stress conferred by H_2_O_2_ and NPS.

### *VdSsk1* Is Required for Melanin Biosynthesis During Microsclerotia Development

**Figure 4:**
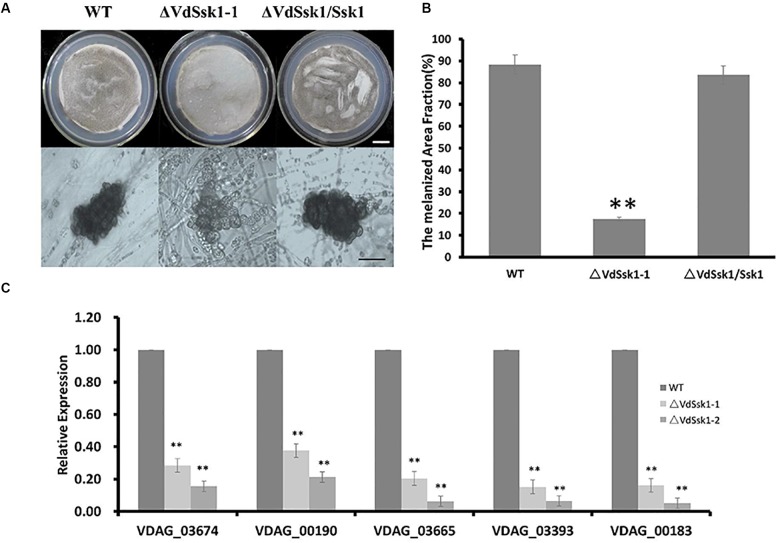
Deletion of VdSsk1 compromises melanin biosynthesis **(A)** conidia at a concretion of 1 × 10^5^/ml of each of the wild type, Δ*VdSsk1-1*, and Δ*VdSsk1/Ssk1* strains, were spread onto cellulose membrane on top of BM agar and allowed to incubate for 6 days. Scale bar = 1.5 cm. **(B)** The melanized area of the colonies was assessed using ImageJ software. Error bars represent the standard deviation of three replicates and two asterisks represent statistically significant differences (*P* < 0.0) revealed by Student’s *t*-test. **(C)** The relative expression level of five genes related to melanin biosynthesis in *V. dahliae*. The expression was normalized against the expression of the *V. dahliae* β-tubulin. Error bars indicate standard deviations from three independent experiments. Statistical significance (^∗∗^*P* < 0.01) was revealed by Student’s *t*-test compared the wild type strain.

To examine whether the Δ*VdSsk1* strain was defective in melanized microsclerotia production, we observed microsclerotia formation on BM. After incubation on BM for 7 days, the Δ*VdSsk1* strain was reduced in dark pigmentation (Figure 4A,B). At 30 days, there remained less melanin in the Δ*VdSsk1* strain (Supplementary Figure S4). Microscopic analyses revealed that the Δ*VdSsk1* strain produced microsclerotia similarly to XS11, but these microsclerotia were devoid of the dark melanization found in the microsclerotia of XS11 and the complemented strain (Figure 4A), indicating that VdSsk1 is dispensable for microsclerotia formation but critical for melanin deposition during microsclerotial maturation.

To further investigate the mechanism of melanin biosynthesis regulated by *VdSsk1*, the five genes (*VDAG*_*03674*, *VDAG*_*00190*, *VDAG*_*03665*, *VDAG*_*03393*, and *VDAG*_*00183*) with known roles in melanin biosynthesis (Wang et al., 2018) were analyzed by transcriptional analysis. Consistent with reduced melanin accumulation in the Δ*VdSsk1* strain, transcripts of all five genes were significantly downregulated in the Δ*VdSsk1* strain compared to those in XS11 (Figure 4C). These data suggested that VdSsk1 positively regulates melanin biosynthesis but not microsclerotia development.

### VdSsk1 Is Required for Full Virulence

**Figure 5:**
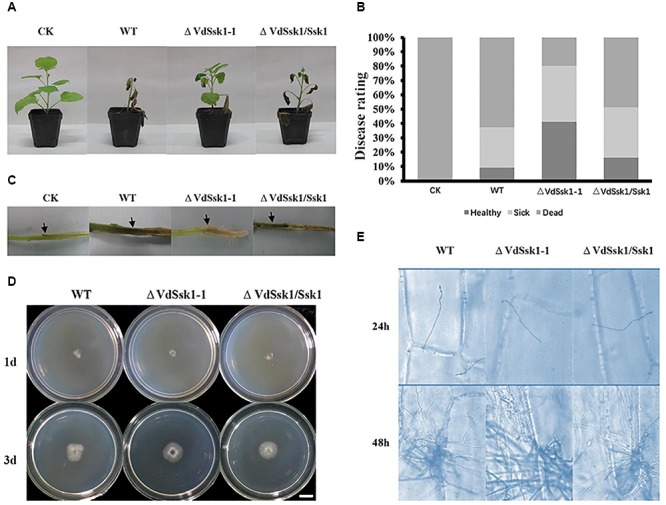
VdSsk1 is essential in full virulence. **(A)** Tabacco seedlings were inoculated with 10^5^ conidial/ml suspension of the wild type, Δ*VdSsk1-1* and Δ*VdSsk1/Ssk1* strains by root-dipping. The control (CK) was mock-inoculated with water. All seedlings were placed in a greenhouse. The typical symptoms were photographed at 35 days after inoculation. **(B)** The chart shows the percentage based on disease rating (healthy, sick, and dead). **(C)** Vascular discoloration symptoms of tobacco stem were observed by longitudinal sectioning. **(D)** All the strains were grown on cellophane for 3 days and the photographs were taken 1 or 3 days after removal of the cellophane membrane. Scale bar = 1 cm. **(E)** Infection assays of onion epidermis examined at 24 and 48 h post inoculation. Fungal hyphae were stained with trypan blue solution.

Compared to XS11 and the Δ*VdSsk1*/*VdSsk1* strain, disruption of *VdSsk1* severely attenuated fungal virulence on tobacco seedlings (Figure 5A,B). The seedlings inoculated by root-dip with conidial suspensions of the Δ*VdSsk1* strain showed slight chlorosis and less wilt, whereas tobacco seedlings inoculated with XS11 and the Δ*VdSsk1/VdSsk1* strain showed obvious wilt symptom or death (Figure 5A,B). At 35 days, the disease rating was significantly lower for the Δ*VdSsk1* strain-inoculated plants than the plants inoculated with XS11 and the complemented strains. Observations of vascular discoloration, a typical symptom of Verticillium wilt (Klosterman et al., 2009), revealed that the Δ*VdSsk1* strain caused only a slight discoloration in plants, however, dark discoloration was apparent in plants infected with the XS11 and the Δ*VdSsk1*/*VdSsk1* strain (Figure 5C).

We further examined the characteristics associated with the infection of the Δ*VdSsk1* strain on a cellophane membrane. The results showed that the Δ*VdSsk1* strain exhibited hyphal penetration of a cellophane membrane similarly to XS11 and the Δ*VdSsk1*/*VdSsk1* strain (Figure 5D). In addition, the formation of hyphopodia was similar among each strain (data not shown). A penetration assay on onion epidermis also revealed that deletion of *VdSsk1* did not affect penetration of invasive hyphae and proliferation within plant cell (Figure 5E). Together, these data suggest that while VdSsk1 is required full virulence, the reduction of virulence of the Δ*VdSsk1* strain is not because of reduced penetration and proliferation into a plant cell.

### VdSsk1 Contributes to Phosphorylation of VdHog1

**Figure 6:**
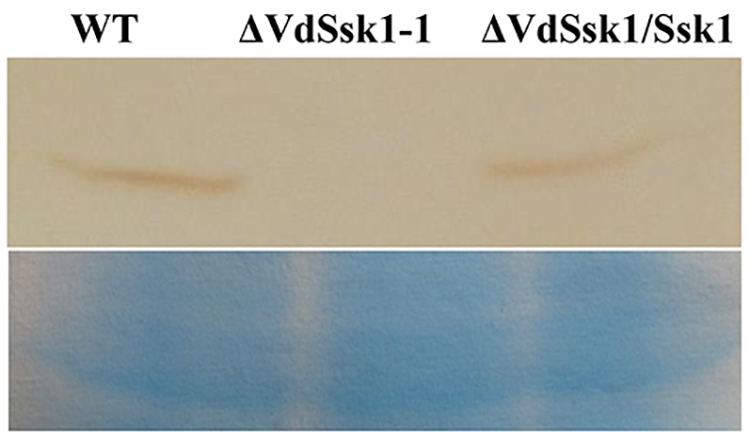
VdHog1 phosphorylation requires VdSsk1 under the osmotic treatment. The phosphorylation levels are shown for VdHog1 in the wild type, Δ*VdSsk1*, and Δ*VdSsk1/Ssk1* strain. All strains were incubated for 3 days in YEPD broth and transferred for 30 min transferred to 1.2 M sorbitol for 30 min. The PAGE-separated total proteins from *V. dahliae* were transferred to a nitrocellulose membrane and probed with the anti-phospho-p38 monoclonal antibody (P-Hog1, Cell Signaling) to detect the phosphorylated form of the VdHog1p via chemiluminescence. The loading control visualized by staining with Coomassie brilliant blue.

To address whether VdSsk1 acts as an upstream of HOG-MAPK pathway, we examined the phosphorylation level of VdHog1 in the Δ*VdSsk1* strain. VdHog1 phosphorylation was not observed in the Δ*VdSsk1* strain (Figure 6). These results suggested that VdSsk1 functions as the upstream activator of the HOG pathway in *V. dahliae*.

## Discussion

To adapt to adverse conditions and potentially harmful surroundings, fungi have evolved sophisticated regulatory networks that confer resistance and adaption to diverse stresses. As shown herein, the RR gene VdSsk1 has important roles in stress response in *V. dahliae*. Additionally, we demonstrated that VdSsk1 is required for melanin biosynthesis and full virulence. Combined with our previous work (Tang et al., 2017), these results provided valuable clues on TCS function in stress responses in *V. dahliae*.

Two RRs of TCS in yeast include Ssk1 and Skn7, components of a signal transduction system that governs cellular responses to various stressful conditions (Stock et al., 2000). To date, Ssk1 is dephosphorylated under osmotic stress to activate HOG MAPK cascade for cell adaptation to osmotic stress. Another RR, Skn7 contains a DNA binding motif and is involved in cellular adaptation to oxidative and thermal stresses. Several previous studies have shown that fungal RR proteins play distinct functions. In *V. dahliae*, VdSkn7 mediates responses to heat shock, cell wall perturbing agents, H_2_O_2_, conidiation, and microsclerotia formation (Tang et al., 2017), while VdSsk1 participates in osmotic and nitrooxidative stress, fungicide resistance, and melanin biosynthesis. Similarly, in *Candida lusitaniae*, Ssk1 is involved in osmotic resistance and pseudohyphal development, whereas Skn7 is crucial for response to oxidative stress (Ruprich-Robert et al., 2008). In *Neurospora crassa*, RRG-1 governs asexual and protoperithecial development, hyperosmotic sensitivity and fungicide resistance; however, RG-2 participates only in the oxidative stress response (Banno et al., 2007; Jones et al., 2007). In other fungi, both Ssk1 and Skn7 are required for development, osmotolerance, and fungicide resistance. For instance, in *Cochliobolus heterostrophus*, both ChSsk1 and ChSkn7 play roles in osmoadaptation and sensitivity to the phenylpyrrole fungicide fludioxonil (Izumitsu et al., 2007). In the present study, we also demonstrated that VdSsk1 positively regulates phosphorylation of VdHog1. We showed that the VdSsk1 deletion exhibited much similar phenotype, including in its response to high osmolarity and fungicide tolerances. Previous studies have shown that deletion of VdPbs2 and VdHog1 results in elevated resistance to iprodione and fludioxonil (Tian et al., 2016; Wang et al., 2016). These data suggest that VdSsk1 functions as an osmotic stress and fungicide resistance regulator mainly via HOG MAPK cascade in *V. dahliae*. In addition to its reduced tolerance to H_2_O_2_, the Δ*VdSsk1* strain also exhibited higher sensitivity to NPS. NO is a by-product of nitrate metabolism and reacts with superoxide anion to generate RNS (Marcos et al., 2015). Unexpectedly, the growth of the Δ*VdSsk1* strain was similar to that of the wild type XS11. How VdSsk1 contributes to the resistance to nitrooxidative stress is not currently known.

Previously, we demonstrated that the Δ*VdSkn7* strain is compromised in its ability to penetrate the plant (Tang et al., 2017). In this study, the Δ*VdSsk1* strain exhibited reduced virulence but we noted that the Δ*VdSsk1* strain was not compromised in its ability to penetrate the plant surface or to form hyphopodia on cellophane. These results indicate that while both of these RRs are involved in the pathogenicity of *V. dahliae*, each may contribute to virulence through select RR signaling routes. Currently, the involvement of Ssk1 and Skn7 in fungal virulence has been reported in phytopathogenic fungi, such as *Magnaporthe oryzae, Fusarium graminearum*, and *Botrytis cinerea*. The deletion of Ssk1 (BRRG-1) results in no obvious changes in virulence in *B. cinerea* (Yan et al., 2011), while the loss of BcSkn7 is impaired (Viefhues et al., 2015). However, in *M. oryzae* and *F. graminearum*, Ssk1 but not Skn7 is shown to be important for virulence (Motoyama et al., 2008; Jiang et al., 2011). These data suggest that the role of RRs in regulating virulence of phytopathogenic fungi varies significantly. Considering data indicating a lack of importance of VdSsk1 in penetration, we thus speculate that VdSsk1 may be involved in the regulation of adaption to xylem niche of the vascular system in plants. Investigating how VdSsk1 mediates vascular adaption and clarifying its specific regulatory mechanism would thus be very interesting. On the other hand, melanin-deficient mutants are commonly characterized as non-pathogenic or as reduced in virulence, even though we have demonstrated that melanin itself is not a virulence factor in *V. dahliae* (Wang et al., 2018). Nevertheless, the Δ*VdSsk1* strain exhibited a significant decrease in melanin production, and therefore it is probable that metabolic or signaling pathways affected by melanin-deficiency contributes to reduced virulence in this strain.

Melanized microsclerotia are critically important in the life cycle and disease spread of *V. dahliae.* Mature microsclerotia are have a thickened cell wall and heavy melanin deposition but we have established that microsclerotia production is independent of melanin deposition since deletion of VdCmr1 eliminates melanin biosynthesis detectable via microscopy but does not affect microsclerotia production (Wang et al., 2018). In addition, we demonstrated that VdCmr1 is regulated by the HOG MAPK pathway. Here, the Δ*VdSsk1* strain produced microsclerotia similar to that of XS11, whereas it also exhibited obvious reductions in melanin biosynthesis. Furthermore, five genes associated with melanin biosynthesis were downregulated in the Δ*VdSsk1* strain. These results suggest that this TCS in *V. dahliae*, which interacts via the HOG pathway also plays an important role in melanin production. However, it is still unknown how or if VdSsk1 interacts with transcription factors such VdCmr1, and how VdSsk1 may govern melanized microsclerotia production through HOG signaling.

## Author Contributions

YW designed the experiments and revised the manuscript. JZ, CT, and CD performed the experiments and analyzed the data. JZ and YW wrote the draft.

## Conflict of Interest Statement

The authors declare that the research was conducted in the absence of any commercial or financial relationships that could be construed as a potential conflict of interest.

## Footnotes

1 https://genome.jgi.doe.gov/Verda1/Verda1.home.html

## Supplementary Material

FIGURE S1 The phylogenetic analysis of VdSsk1 from *V. dahiae* and related orthologs. The phylogenetic relationship between VdSsk1 and other orthologs was constructed by neighbor-joining with 1000 bootstrap replicates, using MEGA version 6.0. The sequences used in the analyses were all acquired from NCBI database.

FIGURE S2 Deletion of VdSsk1 in *V. dahliae*. **(A)** Schematic representation of strategy to disrupt *VdSsk1*. **(B)** PCR assays for identification of gene deletion mutants. **(C)** Southern blotting analyses of the deletion mutants.

FIGURE S3 VdSsk1 disruption does not affect fungal growth on CM with NO_3_^-^ and NO_2_^-^ as a sole nitrogen source. **(A)** Images of various strains cultured on complete medium (CM) contained NH_4_^+^, NO_2_^-^ and NO_3_^-^ for 12 days. Scale bar = 1 cm. **(B)** The chart shows the relative expression of different fungi. Error bars represent the standard deviation of three replicates.

FIGURE S4 VdSsk1 is required for microsclerotia development. The conidia of the wild type, Δ*VdSsk1-1*, and Δ*VdSsk1/Ssk1* strains were each diluted to 1 × 10^5^ conidia/ml and spread on a cellulose membrane overlaid onto basal media (BM) plates or placed on a slide glass for 90 days. Scale bar = 1.5 cm.
