# Supplementary figures and images for: Involvement of a Response Regulator VdSsk1 in Stress Response, Melanin Biosynthesis and Full Virulence in Verticillium dahliae
Source: Front Microbiol. 2019 Mar 22;10:606. doi: 10.3389/fmicb.2019.00606 (PMC6439524; doi:10.3389/fmicb.2019.00606)

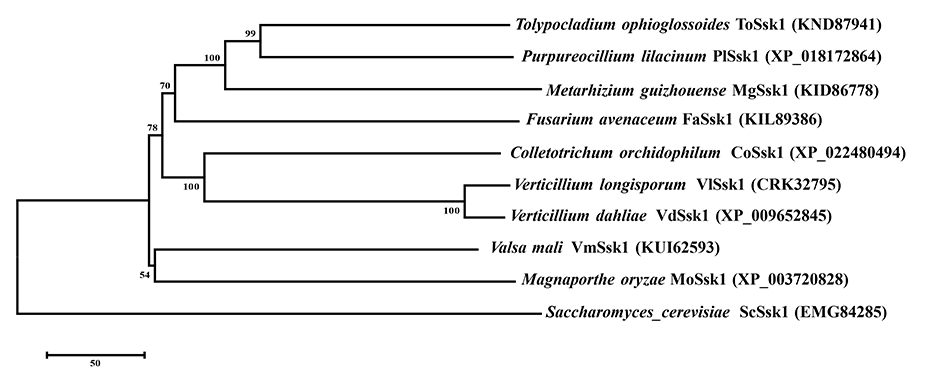

Supplement: FIGURE S1 — The phylogenetic analysis of VdSsk1 from V. dahiae and related orthologs. The phylogenetic relationship between VdSsk1 and other orthologs was constructed by neighbor-joining with 1000 bootstrap replicates, using MEGA version 6.0. The sequences used in the analyses were all acquired from NCBI database. [file Image_1.TIF]

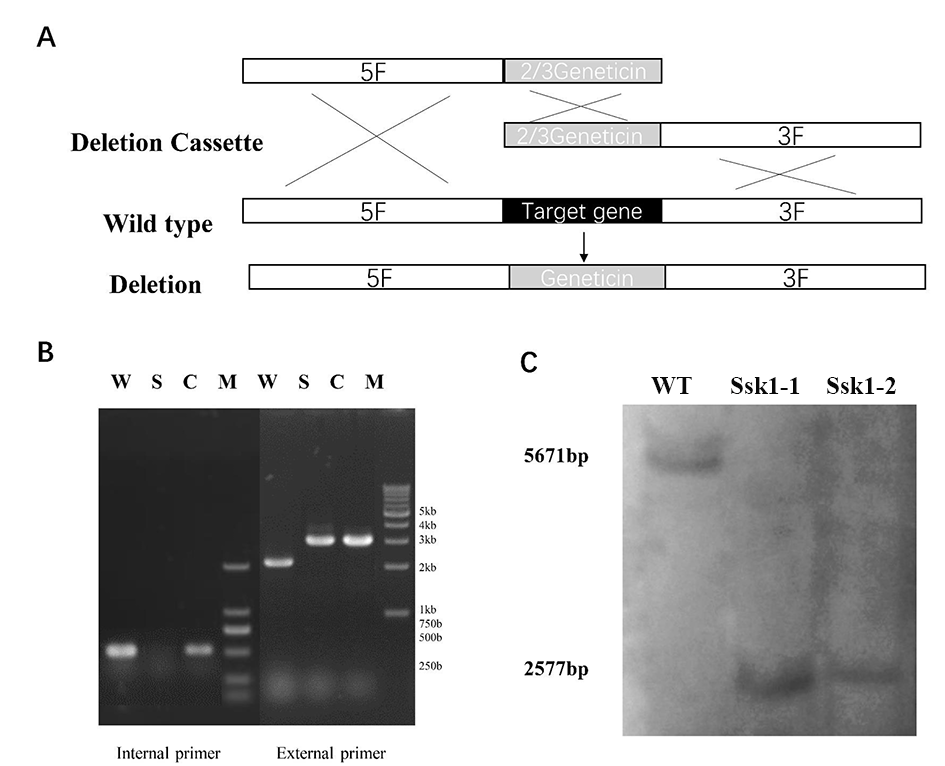

Supplement: FIGURE S2 — Deletion of VdSsk1 in V. dahliae. (A) Schematic representation of strategy to disrupt VdSsk1. (B) PCR assays for identification of gene deletion mutants. (C) Southern blotting analyses of the deletion mutants. [file Image_2.TIF]

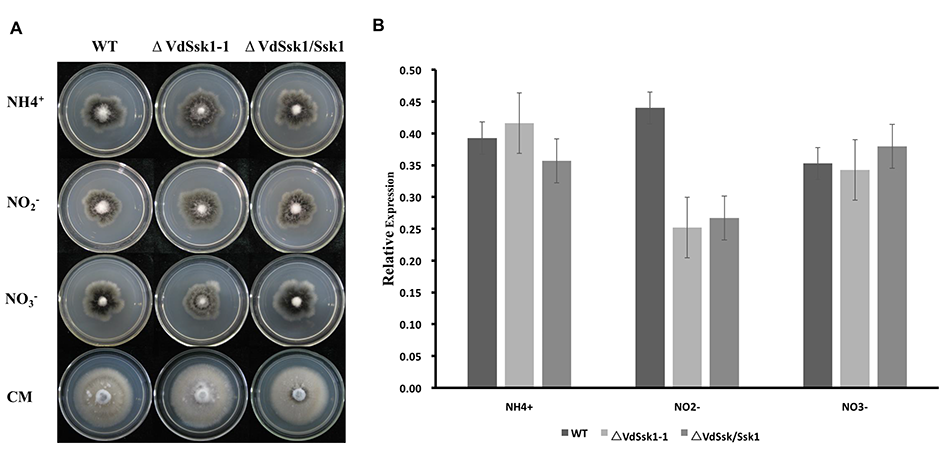

Supplement: FIGURE S3 — VdSsk1 disruption does not affect fungal growth on CM with NO3- and NO2- as a sole nitrogen source. (A) Images of various strains cultured on complete medium (CM) contained NH4+, NO2- and NO3- for 12 days. Scale bar = 1 cm. (B) The chart shows the relative expression of different fungi. Error bars represent the standard deviation of three replicates. [file Image_3.TIF]

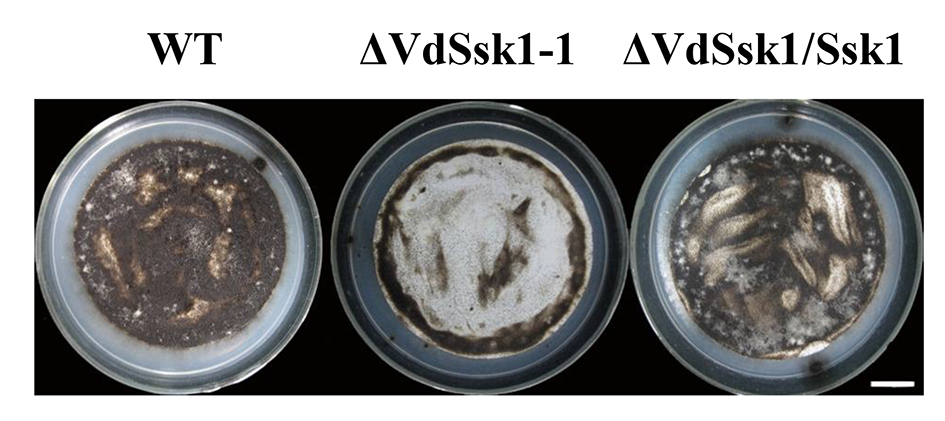

Supplement: FIGURE S4 — VdSsk1 is required for microsclerotia development. The conidia of the wild type, ΔVdSsk1-1, and ΔVdSsk1/Ssk1 strains were each diluted to 1 × 10^5^ conidia/ml and spread on a cellulose membrane overlaid onto basal media (BM) plates or placed on a slide glass for 90 days. Scale bar = 1.5 cm. [file Image_4.TIF]
