# Supplementary material for: Involvement of a Response Regulator VdSsk1 in Stress Response, Melanin Biosynthesis and Full Virulence in Verticillium dahliae
Source: Front Microbiol. 2019 Mar 22;10:606. doi: 10.3389/fmicb.2019.00606 (PMC6439524; doi:10.3389/fmicb.2019.00606)
Supplement: Supplementary file 5 [file Table_1.DOCX]

**Supplementary Table 1 PCR primers used in this study**

| Prime name | Sequence | Description |
| --- | --- | --- |
| Ssk1-5Ffor | GGCCGATGTGTAGACTGGTT | 5F flanking sequence |
| Ssk1-5Frev | AACCGATGGACAAACGTAGC |  |
| Ssk1-3Ffor | ATTGATTTCGACGGATGGAG | 3F flanking sequence |
| Ssk1-3Frev | CGATAGCTTTTGTTGCGTGA |  |
| Ssk1-compfor | CCGATGTGTAGACTGGTTC | complementation |
| Ssk1-comprev | AGGTGTGGAGGTTGAATTG |  |
| Ge-F | GACGTTAACTGATATTGAAGGA | geneticin resistant cassett |
| Ge-R | GCTGGTGACGGAATTTTCAT |  |
| VDAG_03674-F | CAAGTCGATCGGCATGAAGG | qRT-PCR of VDAG_03674 |
| VDAG_03674-R | CACCAACCCCATCTCTCACT |  |
| VDAG_00190-F | CTCGTCCTGATCCGTATCCCA | qRT-PCR of VDAG_00190 |
| VDAG_00190-R | TGTGAATTGAGGCAGGCATG |  |
| VDAG_03665-F | TGGCATCAAGACAGACATGTA | qRT-PCR of VDAG_03665 |
| VDAG_03665-R | ACAACGCGAGCGATGTCGAT |  |
| VDAG_03393-F | AAAGGTGTTTGAGAGCGGAC | qRT-PCR of VDAG_03393 |
| VDAG_03393-R | ATCTCCCTCTCCACAACAGC |  |
| VDAG_00183-F | TATGTCCCTGGCGGCTTTAA | qRT-PCR of VDAG_00183 |
| VDAG_00183-R | TGATCCACTCGCAGTCTTCA |  |
| VdBt-up | AGCTCACCCAGCAGATGTTC | qRT-PCR of β-tubilin |
| VdBt-down | TCGACCTCCTTCATGGCAAC |  |
| Ssk1-s-F | AGGTGTGGAGGTTGAATTG | Probe of southern blot |
| Ssk1-s-R | CGATAGCTTTTGTTGCGTGA |  |
| Ssk1-Infor | ACACTCCTCGTTCCATCC | Mutant sceening |
| Ssk1-Inrew | GCTCACTCGTCACTGATC |  |
| Ssk2-Exfor | TCTGCTCCTGCTCCTATC | Mutant sceening |
| Ssk2-Exrew | AGTCTTGACATTGCGTAGTA |  |
